# Health Anxiety Predicts the Perceived Dangerousness of COVID-19 over and above Intrusive Illness-Related Thoughts, Contamination Symptoms, and State and Trait Negative Affect

**DOI:** 10.3390/ijerph18041933

**Published:** 2021-02-17

**Authors:** Claudio Sica, Corrado Caudek, Silvia Cerea, Ilaria Colpizzi, Maria Caruso, Paolo Giulini, Gioia Bottesi

**Affiliations:** 1 Department of Health Sciences, Psychology Section, University of Firenze, Via San Salvi, 12, 50135 Firenze, Italy; claudio.sica@unifi.it (C.S.); ilaria.colpizzi@unifi.it (I.C.); mcaruso260@gmail.com (M.C.); paolo.guglielmo.giulini@gmail.com (P.G.); 2 Department of Neurosciences, Psychology, Drug Research, and Child Health, University of Firenze, Via San Salvi, 12, 50135 Firenze, Italy; corrado.caudek@unifi.it; 3 Department of General Psychology, University of Padova, Via Venezia, 8, 35131 Padova, Italy; silvia.cerea@unipd.it

**Keywords:** health anxiety, intrusive thoughts, contamination, negative affect, pandemic, psychopathology

## Abstract

This study sought to evaluate the specificity of health anxiety, relative to other forms of psychopathology, in perceptions of COVID-19 as dangerous. Measures of health anxiety, COVID-19 perceived dangerousness, negative affect, anxiety, depression, stress, contamination-related obsessions and compulsions, and intrusive illness-related thoughts were administered online to 742 community individuals during the Italian national lockdown. Results showed that, after controlling for demographic variables and other internalizing problems, health anxiety was the single most important factor associated with the perceived dangerousness of COVID-19. Moreover, a comparison between the current sample’s scores on various symptom measures and scores from prepandemic Italian samples revealed that, whereas other internalizing symptoms increased by a large or very large magnitude during the pandemic, levels of health anxiety and negative affect increased by a medium amount. This result may indicate that health anxiety is relatively trait-like, increasing the likelihood that our correlational data support the model of health anxiety as a vulnerability rather than an outcome. Together, these results indicate that health anxiety may be a specific risk factor for COVID-related maladjustment and support the distinction of health anxiety from other psychological problems.

## 1. Introduction

In late 2019, a respiratory syndrome called coronavirus disease (COVID-19) began to spread, posing a mortal threat to the health of people around the globe [1,2]. Generally, COVID-19 has an incubation period of 1–14 days, and its symptoms include mild to severe fever, cough, dyspnea, and pneumonia [3,4]. The fatality rate is between 1% and 2%. The World Health Organization classified COVID-19 as a pandemic in March 2020. Due to a sharp increase in the number of confirmed cases and deaths, many governments around the world declared a state of emergency and advised people to practice social distancing to minimize contact with others, including self-isolating at home [1,2,5].

Beyond the impact on physical health, ongoing uncertainty related to the pandemic and the dramatic changes in behavior required by social distancing efforts may uniquely and profoundly impact mental health, and these problems may be more likely among individuals with certain psychological conditions [6,7,8]. Specifically, pre-existing health anxiety (although recently changed to “illness anxiety” in the fifth edition of the Diagnostic and Statistical Manual of Mental Disorders [9], “health anxiety” is still in common use by mental health researchers and clinicians and is the term used in the ubiquitous cognitive behavioral model of health anxiety (for a discussion, see Bailer et al. [10])) may represent an important vulnerability factor contributing to heightened concerns about the COVID-19 pandemic. The essential feature of health anxiety, according to the Diagnostic and Statistical Manual of Mental Disorders, fifth edition (DSM-5) [9], is the presence of worries, concerns, or fears of having or acquiring a serious physical disease or other health-related issues [11,12]. Individuals with health anxiety are extremely preoccupied with bodily sensations and functions and with anything that may appear to be a sign of a pathological condition. They may excessively scrutinize medical and health information and frequently look up symptoms and diseases on the Internet (known as cyberchondria) [13,14]. This behavior may lead them to misinterpret trivial symptoms as reflecting serious ailments [15,16].

The aims of the current study were (1) to investigate how health anxiety, relative to other clinical problems, is associated with perceptions of COVID-19 dangerousness and (2) to determine whether the onset of COVID-19 has influenced the prevalence of health anxiety.

### 1.1. Health Anxiety and Epidemics

Few studies have been carried out to evaluate the associations between health anxiety and the fear of infection during an epidemic. This is a surprising limitation, given that one might expect the general tendency toward health-related worries to be associated with heightened concern in the context of disease outbreaks [15]. Blakey and Abramowitz [17] investigated psychological predictors (including health anxiety) of virus-related anxiety in 216 adults during the 2015–2016 Zika outbreak. Overestimations of the likelihood of contamination and greater factual knowledge about Zika emerged as the only variables predicting Zika-related anxiety. Wheaton and colleagues [18] examined the psychological processes associated with swine-flu-related anxiety in 315 college students during the H1N1 influenza pandemic of 2009–2010. Regression analysis indicated that health anxiety symptoms were the third significant predictor (*β* = 0.21) of swine-flu-related anxiety, after contamination fears and disgust sensitivity (both *βs* = 0.28).

More recently, Jungmann and Witthöft [19] conducted an online survey with 1615 individuals to investigate the roles of health anxiety, cyberchondria, and coping in COVID-19-related anxiety. Health anxiety showed positive relationships with virus anxiety (*r* = 0.34), distress caused by Internet research (*r* = 0.48), and maladaptive emotion regulation (*r* = 0.17). In addition, individuals with heightened health anxiety reported an increase in virus-related anxiety in recent months, according to a retrospective report. Importantly, however, findings from this last study do not allow for firm conclusions to be drawn about the specificity of the relationship between health anxiety and COVID-19-related anxiety, since other potentially relevant psychological variables—such as depression, contamination compulsions, and general anxiety—were not taken into account.

Similarly, Cannito and colleagues [20] found that during the national lockdown in Italy, health anxiety predicted attentional bias toward virus-related objects [21]. However, as in the study above, it was not possible to rule out the influence of general psychological distress or other clinical variables. Indeed, attentional bias toward threats is a common phenomenon among anxious populations [22], so it is unclear if health anxiety plays a specific role in disease-related cognitive processing.

### 1.2. The Current Study

The current study sought to extend prior research on health anxiety during disease outbreaks through the following main aims:

(1) To clarify the specific role of health anxiety in disease-related cognition, over and above other forms of psychopathology. Because health-related worries occur in other psychological disorders beyond health anxiety [23,24,25,26,27,28], it is not clear to what extent health anxiety symptoms contribute to perceptions of COVID-19 as dangerous, over and above general distress and symptoms of generalized anxiety disorder, obsessive-compulsive disorder, and depressive disorders. In addition, during a disease outbreak, transient illness-related intrusive thoughts are fairly common [29,30] and do not necessarily indicate the presence of clinical health anxiety. Therefore, we also wanted to rule out the possibility that the purported link between health anxiety and perceptions of COVID-19 dangerousness were driven by these transient thoughts.

(2) To compare levels of health anxiety during the COVID-19 pandemic to prepandemic statistics. The literature suggests that health anxiety is relatively chronic, but it may also fluctuate in relation to life events [31]. Therefore, we also hoped to ascertain whether, on average, people reported more health anxiety symptoms during the pandemic than in pandemic-free periods, suggesting a prominent effect of stressful life events, or if reported health anxiety symptoms remained consistent, suggesting a more stable course.

Drawing on the scarce extant literature, the following hypotheses were tested: (1) health anxiety, negative affect, contamination compulsions, generalized anxiety, depression symptoms, and intrusive illness-related thoughts should be all related to the perceived dangerousness of COVID-19, and (2) health anxiety should be uniquely associated with perceived dangerousness of COVID-19, over and above the other psychological variables. Because of the variability of the prior literature, we had no a priori hypotheses about how the level of health anxiety reported by our participants would compare to prepandemic levels in similar samples.

## 2. Materials and Methods

### 2.1. Participants and Procedures

Data for the current study were collected in Italy during the period of maximal national restrictions in response to COVID-19 (i.e., from 10 March 2020 to 2 June 2020). An online battery of questionnaires was advertised through social media platforms (Facebook, Twitter, and Instagram). There were no exclusionary criteria, and the online battery took about one hour to complete. Of note, the sample consisted of community members and was not selected for elevated health anxiety. We believe this to be a strength, as health anxiety represents a continuum ranging from the absence of health concerns to pathological health anxiety [32,33]. When examining the psychological processes surrounding health anxiety, it is beneficial to use large, unselected samples that include a full range of symptoms, rather than focusing exclusively on samples of individuals with severe levels of health anxiety [10,33].

Ethical approval was obtained from the Institutional Board of the University of Firenze, in accordance with the principles of the Declaration of Helsinki. All participants were informed about the study’s aims and provided informed consent before completing the survey.

### 2.2. Measures

A sociodemographic questionnaire was administered to collect background information such as sex, age, level of education, marital status, and place of residence. Participants were also asked if they, a close family member, or a significant other had contracted COVID-19.

The Perceived Dangerousness of Infection Questionnaire (PDIQ) was developed for the purposes of the current study to assess participants’ perceptions of the dangerousness of COVID-19. Items were designed to assess participants’ perceptions of the extent of the threat posed by COVID-19, including both the likelihood they would contract COVID-19 and the anticipated degree of personal harm an infection would cause [34]. As a first step, a pool of 10 items was collaboratively developed by a group of clinicians and researchers with experience in evaluating and treating individuals with anxiety disorders and somatization. Next, 30 individuals from the community rated the degree of intelligibility and clarity of the provisional items, using two separate five-point Likert scales ranging from 0 (“poor”) to 4 (“excellent”). Comments by each participant about the items were also recorded. Only the items that received a mean rating of 3 or higher for both intelligibility and clarity were included in the final questionnaire. The final version of the PDIQ comprised nine items, which participants rated on a Likert scale ranging from 1 (“I do not agree at all”) to 4 (“I fully agree”). Sample items included, “When I think of Coronavirus, I feel much more nervous than usual” and “I don’t understand why people care so much about Coronavirus”. A total score was created by reversing items keyed in the direction of low dangerousness, such that a higher total score indicated elevated perception of the dangerousness of COVID-19. In the current sample, internal consistency reliability for the PDIQ total score was acceptable (Cronbach’s *α* = 0.71).

The Health Anxiety Questionnaire (HAQ) [35] is a 21-item questionnaire measuring the main manifestations of health anxiety. Cluster and factor analyses have revealed four factors: worry and health preoccupation, fear of illness and death, reassurance-seeking behavior, and the extent to which symptoms interfere with a person’s life. Prior studies indicate that the HAQ has appropriate reliability and discriminant validity in both the original and the Italian [36] versions. In the current sample, internal consistency for the HAQ total score was excellent (Cronbach’s *α* = 0.93).

To compare health anxiety to other forms of psychopathology in predicting COVID-19 perceptions, we also administered the following self-report instruments:

The Questionnaire of Unpleasant Intrusive Thoughts (QUIT) [37] is a measure assessing cognitive intrusions of various types. The QUIT begins with a detailed definition of unwanted mental intrusions and the different ways they can be experienced (i.e., as images, thoughts/doubts, impulses, or physical sensations). After the initial description, four separate sets of intrusions are presented: obsessional (i.e., related to obsessive-compulsive disorder; 12 items), appearance-related (i.e., related to body dysmorphic disorder; 9 items), illness and death-related (i.e., related to health anxiety; 10 items) and eating-related (i.e., related to eating disorders; 8 items). Respondents are then requested to evaluate each intrusion for frequency from 0 (“never”) to 6 (“always, frequently throughout the day”) and the discomfort it produces when it occurs from 0 (“not at all”) to 4 (“extremely disturbing”). The QUIT was validated in a cross-cultural study [32] carried out in Europe (including Italy), the Middle East, and South America. In the current study, only the discomfort score associated with health anxiety-related intrusions (e.g., “For no particular reason, I have intrusive thoughts such as ‘I could die of a serious illness,’ for example, cancer, AIDS, etc.”) was used, given the high correlation between discomfort and frequency (*r*=0.90). In the current sample, internal consistency for this scale was very good (Cronbach’s *α* = 0.90).

The Obsessive-Compulsive Inventory-Revised (OCI-R) [38] is a widely used 18-item self-report questionnaire measuring the severity of obsessive-compulsive symptoms on a five-point Likert scale. Items are grouped into six subscales (washing/contamination, checking, ordering, obsessing, hoarding, and mental neutralizing). Reliability and validity of this instrument are supported both in the original and in the Italian [39] versions. In the current study, we used the washing/contamination scale only (which was consistently related to pandemic-related problems [17,18]) and the Cronbach’s *α* was 0.70.

The Depression Anxiety Stress Scale-21 (DASS-21) [40] is a 21-item measure assessing depression (lack of incentive, low self-esteem, and dysphoria), anxiety (somatic and subjective symptoms of anxiety as well as acute responses of fear), and stress (irritability, impatience, tension, and persistent arousal) over the previous week on a four-point Likert scale. Good psychometric properties have been reported for both the original and the Italian [41] versions. In the current study, Cronbach’s αs for depression, anxiety, and stress were all above 0.90.

The personality inventory for DSM-5 personality disorders (PID-5) [42] consists of 220 items rated on a four-point Likert scale assessing 25 facet traits that that load onto five higher-order dimensions: antagonism, detachment, disinhibition, negative affect, and psychoticism. The PID-5 has adequate psychometric properties in its original version [43,44] as well as in the Italian translation [45,46]. In the current study, we used the negative affect scale only, and its internal consistency was excellent (Cronbach’s *α* = 0.91). We chose a measure of negative affect as an index of general internalizing psychopathology, since it is thought to subsume most internalizing symptoms, and it is strictly related to neuroticism [47].

### 2.3. Statistical Analyses

Zero-order correlations (Pearson’s *rs*) were computed to evaluate the associations among all study variables. Following Cohen’s classification [48], large correlations were defined as 0.50 and above, medium correlations between 0.30 and 0.49, and small correlations between 0.10 and 0.29. In addition, Steiger’s *z* test was used to evaluate magnitude differences between correlations.

To evaluate the unique association between the HAQ score and the PDIQ (Aim 1), we used a multiple regression analysis. In the first block, age, education, and gender (dummy coded: 1 = males, 2 = females) were entered to control for any effect of demographic variables. In the second block, all the symptom variables that were found to correlate with the PDIQ were entered, apart from the HAQ. In the third and final block, the HAQ score was included. In this way, we were able to evaluate the association between health anxiety and perceived COVID-19 dangerousness, over and above the other psychopathology variables.

To address Aim 2, independent-samples *t*-tests were run to compare the average scores on each symptom measure in our sample, collected during the COVID-19 lockdown, versus the previously published Italian validation samples (i.e., pre- versus peri-COVID-19 scores). Hedges**’** *g* coefficients were computed to evaluate the effect size of the differences. These effects are considered small at or below 0.2, medium around 0.5, and large above 0.8 [48]. All the statistical analyses were conducted using IBM SPSS, version 26.

## 3. Results

Of the 743 adults who enrolled in the study, 742 (99.8%) completed all questionnaires. The mean age of this final sample was 30.7 years (SD = 14.0), and 73% was female. Their mean education was 14.4 years (SD = 3.5); 69% of the sample was single, 26% was married or cohabitating, and 4% was separated or divorced. Geographically, 23% lived in Northern Italy, 65% in Central Italy, and 12% in Southern Italy. None of the participants reported being ill or infected by COVID-19 themselves, but 65 (8.7%) reported that a close family member or significant other had contracted the virus.

Bivariate correlations among all study variables are presented in Table 1.

All the variables were significantly related to the PDIQ at a small magnitude, except for the DASS-21 Depression scale, which showed a negligible correlation coefficient. In turn, the HAQ was significantly associated with all the other variables at a small size, except for the QUIT Health Discomfort score (*r* = 0.41, *p* < 0.001) and PID-5 Negative Affect scale (*r* = 0.40, *p* < 0.001). According to the Steiger’s *z* test, the latter two correlation coefficients were significantly larger than the correlations between the HAQ and all the other variables (*p* < 0.001). Unsurprisingly, the PID-5 Negative Affect score appeared, on average, as the largest association with all the other variables (mean *r* = 0.36).

Findings from the multiple regression analysis are shown in Table 2. Because DASS-21 Depression was not correlated with PDIQ scores, it was not included in the model. Inspection of the final model indicated that multicollinearity was not a problem [49].

Results showed that each successive step of the regression added significantly to the overall prediction of PDIQ scores (ΔR^2^). In the final model, female gender, younger age, the OCI-R Washing/Contamination scale, and the HAQ score were the only significant predictors of the PDIQ. Overall, the final model explained 12% of the variance in PDIQ scores; the HAQ explained 4% of the variance in PDIQ score beyond that explained by the other variables.

Lastly, we compared the average scores on all questionnaires completed by the current sample with the normative values reported in the Italian standardization studies (Table 3).

Results showed that all symptom scores were significantly higher in the current COVID-19 sample than in the pre-COVID-19 Italian validation samples, except for QUIT Health Discomfort scores, which were surprisingly significantly lower than in the pre-COVID-19 sample. Hedges’ *g* was medium-sized for HAQ, QUIT, and PID-5 Negative Affect scores and large for OCI-R Washing/Contamination and each of the DASS-21 scale scores.

## 4. Discussion

Correlational findings showed that all the variables examined in this study were relevant to the perceived dangerousness of COVID-19, except for depression. Depression may be more closely related to the consequences of the pandemic (e.g., living in quarantine) than concerns about its dangerousness [50,51]. Consistent with the high comorbidity of health anxiety [10], different psychopathological symptoms were significantly linked to HAQ scores. For example, illness-related intrusions were moderately associated with health anxiety, demonstrating that these two phenomena are related but not overlapping. While transient intrusive thoughts about illness and its consequences may occur in any individual during a pandemic, excessive preoccupation and concern about one’s health—which are characteristic of health anxiety and reflected in HAQ scores—appear more relevant to perceptions of COVID-19 dangerousness.

Regression results indicated that health anxiety, as measured by the HAQ, was the single most important factor associated to the perceived dangerousness of COVID-19. This result is an important step beyond the existing literature given that, in this study, other relevant psychopathological variables were taken in account. Even though recent studies have stressed the role of general tendencies toward health anxiety in COVID-19-related concerns [19], this is one of the first studies demonstrating a specific association between health anxiety and the perceived dangerousness of COVID-19, over and above other forms of internalizing.

In addition to health anxiety, the present study suggests that individuals with obsessive-compulsive symptoms related to washing and contamination may also be sensitive and vulnerable to COVID-19 fears. This finding is consistent with the literature and suggests that these individuals may be at risk of exacerbation of obsessive-compulsive symptoms during COVID-19 [52,53]. Importantly, this vulnerability appears independent from health anxiety, given that our regression analyses elucidated the *unique* contributions of each variable of interest to perceptions of COVID-19 dangerousness.

Regarding demographic variables, younger individuals and females appeared to be more concerned about the dangerousness of COVID-19, indicating that these individuals may be more prone to developing distressing symptoms during a pandemic [51].

The results summarized above suggest that in disease-threat situations, individuals with high levels of health anxiety may react differently than people with low levels of health anxiety. For instance, Höfling and Weck [54] reported that processes such as worries about one’s health, perception of others as unsupportive of the respondent’s illness concerns, tendency toward reassurance-seeking behavior with regard to illness concerns, and preoccupation with bodily sensations were more intense for patients with hypochondriasis in contrast to those with panic disorder or social phobia [55,56]. Consistent with this finding, current theoretical conceptualizations emphasize the importance of cognitive processes in the maintenance of severe health anxiety [14,21,57]. The link between health anxiety and perceptions of COVID-19 as dangerous, as found in the current study, should not be overlooked. For instance, anxiety about becoming ill with COVID-19 could lead people to visit health care facilities excessively and often, thereby increasing the risk of transmission and hindering the provision of necessary medical care to patients in real need. Moreover, individuals who are highly concerned about being infected may undertake excessive or iatrogenic protective measures. In addition, excessive control and reassurance-seeking among people who are overly concerned about their health may place a significant burden on the health care system and trigger socially disruptive behaviors [58].

The unique nature of health anxiety is also demonstrated by the comparison between the scores of our community sample during COVID-19 lockdown with those obtained in similar samples before the pandemic. Whereas symptom scores such as generalized anxiety, stress, depression, and contamination-related intrusive thoughts increased by a large or very large magnitude, HAQ and PID-5 Negative Affect scores increased by a medium amount only. This result may indicate that health anxiety symptoms are approximately as stable as a personality trait like negative affect, even in a crisis context when other clinical symptoms are increasing drastically. In contrast, the distress linked to intrusive thoughts about illness was lower during the pandemic than in the pre-COVID-19 period. This result may be due to the difference between samples, as the QUIT validation sample [37] tested in the prepandemic period included only college students, whereas our community sample had a higher mean age and lower mean educational attainment. Another possible explanation regards habituation mechanisms. Indeed, frequent and inescapable news and government warnings about the infection might have acted as a form of exposure to intrusive thoughts, resulting in less distress. In any case, the contrasting patterns of pre- to peri-COVID scores seem to further demonstrate that intrusive thoughts about illness and health anxiety are qualitatively different phenomena.

The specific role of health anxiety demonstrated in this study adds robustness to the distinction of health anxiety from other psychopathological conditions, as illustrated by the placement of illness anxiety disorder in a separate section named “Somatic Symptom and Related Disorders” in both DSM-5 [9] and in the Psychodynamic Diagnostic Manual [54,59]. Health anxiety has long held an uncertain place in prominent taxonomies of mental illness. This fact is illustrated in recent changes to the Hierarchical Taxonomy of Psychopathology (HiTOP), a quantitative-empirical nosology initiative. Initially, health anxiety was provisionally considered to fall under the Somatoform Spectrum in HiTOP, separate from the other major spectra (i.e., Internalizing, Thought Disorder, Disinhibited Externalizing, Antagonistic Externalizing, and Detachment) [60]. However, in more recent HiTOP studies based on updated structural models, health anxiety is considered a “syndrome” falling under the Somatic Anxiety Sub-Subfactor of the Fear Subfactor, which is contained within Internalizing Spectrum [61]. Given these recent changes, the placement of health anxiety in taxonomies of psychopathology remains to be clarified. Further research on the specific cognitive processes that produce health anxiety and differentiate it from other forms of internalizing could contribute to these efforts [62].

Some study limitations warrant mention. The cross-sectional nature of this investigation precludes us from drawing causal inferences regarding the relationships between the symptom variables and concerns about the COVID-19 pandemic. Nonetheless, the finding of relative stability of health anxiety between pre- and peri-COVID samples, compared with other forms of psychopathology, lends initial support to the theoretical model described here, wherein pre-existing health anxiety makes an individual more likely to perceive COVID-19 as dangerous. Another limitation is that, in the current study, a large portion of variance in the perceived dangerousness of COVID-19 remains unexplained, thereby requiring more research about the factors associated with COVID-19-related perceptions. Future studies using a longitudinal design could consider the System Dynamics approach as a means to model the various influences on perceptions of COVID-19 as dangerous [63,64]. In addition, the use of an Italian sample may limit generalizability to other countries, as results may not be identical for individuals with different backgrounds and pandemic-related stressors or in countries with different government responses. Lastly, given that frequency and/or duration of online health research correlates consistently with health anxiety and often provokes anxiety [19,65], it will be important for future research to investigate the role of the Internet in the association between health anxiety and perceived dangerousness of COVID-19. 

## 5. Conclusions

Notwithstanding the above-mentioned limitations, the present study contributes to the existing literature by demonstrating the specific influence of health anxiety on perceptions of COVID-19 as dangerous. Our results have potential clinical implications. Although our data are correlational, they are consistent with the idea that individuals with health anxiety symptoms could be especially vulnerable to anxiety about ongoing disease threats, especially in the context of ongoing media and governmental advisories to employ stringent precautionary measures. Clinicians can help these individuals to respond to this information in a more adaptive way by challenging their perceptions of the likelihood and severity of infection, thus reducing excessive and pathological fear and avoidance behaviors. In general, cognitive-behavioral therapy components such as psychoeducation, cognitive restructuring, and exposure therapy have shown utility in health anxiety management [31]. Additionally, given the inability to fully avoid aversive and anxiety-provoking information during a global pandemic, acceptance-based approaches such as Acceptance and Commitment Therapy [66] could be employed to increase one’s willingness to experience uncomfortable thoughts and feelings about COVID-19 dangerousness without trying to avoid or struggle with them [67].

## Figures and Tables

**Table 1:** Bivariate correlations (Pearson’s *r*s) among psychopathological variables (*N* = 742).

Measure	1	2	3	4	5	6	7	8
1. PDIQ		0.26 **	0.21 **	0.04	0.13**	0.13**	0.18 **	0.14 **
2. HAQ			0.40 **	0.21 **	0.26 **	0.26**	0.16 **	0.41 **
3. PID-5 Negative Affect				0.46**	0.38 **	0.51**	0.26 **	0.42 **
4. DASS-21 Depression					0.58 **	0.70 **	0.12 **	0.20 **
5. DASS-21 Anxiety						0.67 **	0.21 **	0.27 **
6. DASS-21 Stress							0.15 **	0.30 **
7. OCI-R Washing/Contamination								0.28 **
8. QUIT Health Discomfort								

PDIQ = Perceived Dangerousness of Infection Questionnaire, HAQ = Health Anxiety Questionnaire, PID-5 = Personality Inventory for DSM-5 Personality Disorders, DASS-21 = Depression Anxiety Stress Scale-21, OCI-R = Obsessive-Compulsive Inventory-Revised, and QUIT = Questionnaire of Unpleasant Intrusive Thoughts. ** *p* < 0.001; figures for the HAQ are bolded.

**Table 2:** Results of linear regression analysis predicting Perceived Dangerousness of Infection Questionnaire score.

Predictors	*B*	*SE B*	*β*	*t*	*ΔR2*	*F*	*df1*	*df2*
**Step 1**					0.03 **	8.05	3	738
(Constant)	27.01	0.99		27.16 **				
Age	−0.02	0.01	−0.08	−2.17 *				
Gender	1.34	0.34	0.15	4.00 **				
Education	−0.03	0.05	−0.02	−0.58				
**Step 2**					0.07 **	8.38	8	733
(Constant)	24.59	1.06		23.25 **				
Age	−0.02	0.01	−0.08	−2.07 *				
Gender	1.05	0.33	0.11	3.17 *				
Education	0.03	0.05	0.02	0.60				
PID-5 Negative Affect	0.04	0.01	0.13	2.80 *				
DASS-21 Anxiety	0.02	0.02	0.04	0.91				
DASS-21 Stress	−0.01	0.02	−0.02	−0.33				
OCI-R Washing/Contamination	0.20	0.06	0.14	3.60 **				
QUIT Health Discomfort	0.01	0.02	0.03	0.66				
**Step 3**					0.11 **	10.83	9	732
(Constant)	22.43	1.12		20.09 **				
Age	−0.02	0.01	−0.08	−2.16 *				
Gender	1.11	0.33	0.12	3.42 *				
Education	0.03	0.05	0.02	0.52				
PID-5 Negative Affect	0.02	0.02	0.07	1.54				
DASS-21 Anxiety	0.01	0.02	0.02	0.41				
DASS-21 Stress	−0.00	0.02	−0.01	−0.21				
OCI-R Washing/Contamination	0.20	0.06	0.14	3.67 **				
QUIT Health Discomfort	−0.02	0.02	−0.03	−0.79				
HAQ	0.08	0.02	0.21	5.29 **				

PID-5 = Personality Inventory for DSM-5 Personality Disorders, DASS-21 = Depression Anxiety Stress Scale-21, OCI-R = Obsessive-Compulsive Inventory-Revised, QUIT = Questionnaire of Unpleasant Intrusive Thoughts, and HAQ = Health Anxiety Questionnaire. * *p* < 0.05, ** *p* < 0.001.

**Table 3:** Comparison between the current sample and the original Italian standardization sample on various measures of psychopathology.

	HAQ	QUIT Health Discomfort	OCI-R Washing/Contamination	DASS-21 Depression	DASS-21 Anxiety	DASS-21 Stress	PID-5 Negative Affect
Current sample	39.9 (11.1)	12.6 (9.0)	12 (2.8)	12.9 (10.3)	11.1 (9.3)	18.6 (10.2)	29.9 (12.5)
Pre-COVID sample	33.8 (9.2)	19.5 (9.9)	0.9 (1.5)	3.5 (3.2)	2.4 (2.6)	6.4 (3.8)	23.2 (9.9)
*t*-test outcome	7.8 *	−7.5 *	68.7 *	18.4 *	18.7 *	23.5 *	9.2 *
Hedges’ *g*	0.57	0.75	4.5	1.11	1.14	1.58	0.57

Standard deviation in parentheses. HAQ = Health Anxiety Questionnaire (pre-COVID-19 sample *N* = 252 community individuals [36]), QUIT = Questionnaire of Unpleasant Intrusive Thoughts (pre-COVID-19 sample *N* = 114 undergraduates [37]), OCI-R = Obsessive-Compulsive Inventory-Revised (pre-COVID-19 sample *N* = 340 community individuals [39]), DASS-21 = Depression Anxiety Stress Scale-21 (pre-COVID-19 sample *N* = 417 community individuals [41]), and PID-5 = Personality Inventory for DSM-5 Personality Disorders (pre-COVID-19 sample *N* = 389 community individuals [45]). * *p* < 0.01.

## Data Availability

The data presented in this study are available on request from the corresponding author. The data are not publicly available due to their use in other ongoing works.

## References

[1] World Health Organization (2020). Coronavirus Disease (COVID-19) Pandemic. https://www.who.int/emergencies/diseases/novel-coronavirus-2019.

[2] The Novel Coronavirus Pneumonia Emergency Response Epidemiology Team (2020). The epidemiological characteristics of an outbreak of 2019 novel coronavirus diseases (COVID-19) in China. China CDC Wkly.

[3] Centers for Disease Control and Prevention (2020). About COVID-19. https://www.cdc.gov/coronavirus/2019-ncov/cdcresponse/about-COVID-19.html.

[4] Huang C., Wang Y., Li X., Ren L., Zhao J., Hu Y., Zhang L., Fan G., Xu J., Gu X. (2020). Clinical features of patients infected with 2019 novel coronavirus in Wuhan, China. Lancet.

[5] Kucharski A.J., Russell T.W., Diamond C., Liu Y., Edmunds J., Funk S., Eggo R.M. (2020). Early dynamics of transmission and control of COVID-19: ADS mathematical modelling study. Lancet Infect. Dis..

[6] Cao W., Fang Z., Hou G., Han M., Xu X., Dong J., Zheng J. (2020). The psychological impact of the COVID-19 epidemic on college students in China. Psychiatry Res..

[7] Cénat J.M., Blais-Rochette C., Kokou-Kpolou C.K., Noorishad P.G., Mukunzi J.N., McIntee S.E., Dalexis R.D., Goulet M.A., Labelle P.R. (2020). Prevalence of symptoms of depression, anxiety, insomnia, posttraumatic stress disorder, and psychological distress among populations affected by the COVID-19 pandemic: A systematic review and meta-analysis. Psychiatry Res..

[8] Pfefferbaum B., North C.S. (2020). Mental Health and the Covid-19 Pandemic. N. Engl. J. Med..

[9] American Psychiatric Association (2013). Diagnostic and Statistical Manual of Mental Disorders (DSM-5).

[10] Bailer J., Kerstner T., Witthöft M., Diener C., Mier D., Rist F. (2016). Health anxiety and hypochondriasis in the light of DSM-5. Anxiety Stress Coping.

[11] Tyrer P. (2018). Recent advances in the understanding and treatment of health anxiety. Curr. Psychiatry Rep..

[12] Bobevski I., Clarke D.M., Meadows G. (2016). Health anxiety and its relationship to disability and service use: Findings from a large epidemiological survey. Psychosom. Med..

[13] McMullan R.D., Berle D., Arnáez S., Starcevic V. (2019). The relationships between health anxiety, online health information seeking, and cyberchondria: Systematic review and meta-analysis. J. Affect. Disord..

[14] Witthoft M., Hiller W. (2010). Psychological approaches to origins and treatments of somatoform disorders. Annu. Rev. Clin. Psychol..

[15] Tyrer P. (2020). Why health anxiety needs to be recognised in hospital practice. Clin. Med..

[16] Salkovskis P.M., Warwick H.C., Asmundson G.J.G., Taylor S., Cox B.J. (2001). Making sense of hypochondriasis: A cognitive model of health anxiety. Health Anxiety: Clinical and Research Perspectives on Hypochondriasis and Related Conditions.

[17] Blakey S.M., Abramowitz J.S. (2017). Psychological predictors of health anxiety in response to the Zika virus. J. Clin. Psychol. Med. Settings.

[18] Wheaton M.G., Abramowitz J.S., Berman N.C., Fabricant L.E., Olatunji B.O. (2011). Psychological predictors of anxiety in response to the H1N1 (swine flu) pandemic. Cogn. Ther. Res..

[19] Jungmann S.M., Witthöft M. (2020). Health anxiety, cyberchondria, and coping in the current COVID-19 pandemic: Which factors are related to coronavirus anxiety?. J. Anxiety Disord..

[20] Cannito L., Di Crosta A., Palumbo R., Ceccato I., Anzani S., La Malva P., Di Domenico A. (2020). Health anxiety and attentional bias toward virus-related stimuli during the COVID-19 pandemic. Sci. Rep..

[21] Marcus D.K., Gurley J.R., Marchi M.M., Bauer C. (2007). Cognitive and perceptual variables in hypochondriasis and health anxiety: A systematic review. Clin. Psychol. Rev..

[22] Bar-Haim Y., Lamy D., Pergamin L., Bakermans-Kranenburg M.J., Van Ijzendoorn M.H. (2007). Threat-related attentional bias in anxious and nonanxious individuals: A meta-analytic study. Psychol. Bull..

[23] Asmundson G.J., Abramowitz J.S., Richter A.A., Whedon M. (2010). Health anxiety: Current perspectives and future directions. Curr. Psychiatry Rep..

[24] Olatunji B.O., Deacon B.J., Abramowitz J.S. (2009). Is hypochondriasis an anxiety disorder?. Br. J. Psychiatry.

[25] Sakai R., Nestoriuc Y., Nolido N.V., Barsky A.J. (2010). The prevalence of personality disorders in hypochondriasis. J. Clin. Psychiatry.

[26] Sunderland M., Newby J.M., Andrews G. (2013). Health anxiety in Australia: Prevalence, comorbidity, disability and service use. Br. J. Psychiatry.

[27] Taylor S., McKay D., Abramowitz J.S., Sturmey P., Hersen M. (2012). Hypochondriasis and Health-Related Anxiety. Handbook of Evidence-Based Practice in Clinical Psychology.

[28] McClure E.B., Lilienfeld S.O., Asmundson G.J.G., Taylor S., Cox B.J. (2001). Personality traits and health anxiety. Health Anxiety: Clinical and Research Perspectives on Hypochondriasis and Related Conditions.

[29] Barsky A.J., Fama J.M., Bailey E.D., Ahern D.K. (1998). A Prospective 4- to 5-Year Study of DSM-III-R Hypochondriasis. Arch. Gen. Psychiatry.

[30] Simon G.E., Gureje O., Fullerton C. (2001). Course of hypochondriasis in an international primary care study. Gen. Hosp. Psychiatry.

[31] Taylor S., Asmundson G.J. (2004). Treating Health Anxiety: A Cognitive-Behavioral Approach.

[32] Ferguson E. (2009). A taxometric analysis of health anxiety. Psychol. Med..

[33] Norbye A.D., Abelsen B., Førde O.H., Ringberg U. (2020). Distribution of health anxiety in a general adult population and associations with demographic and social network characteristics. Psychol. Med..

[34] Freeston M.H., Tiplady A., Mawn L., Bottesi G., Thwaites S. (2020). Towards a model of uncertainty distress in the context of Coronavirus (Covid-19). Cogn. Behav. Ther..

[35] Lucock M.P., Morley S. (1996). The health anxiety questionnaire. Br. J. Health Psychol..

[36] Melli G., Coradeschi D., Smurra R. (2007). The Italian version of Health Anxiety Questionnaire: Reliability and factorial analysis. Psicoter. Cogn. Comport..

[37] Pascual-Vera B., Akin B., Belloch A., Bottesi G., Clark D.A., Doron G., Fernández-Alvarez H., Ghisi M., Gómez B., Inozu M. (2019). The cross-cultural and transdiagnostic nature of unwanted mental intrusions. Int. J. Clin. Health Psychol..

[38] Foa E.B., Huppert J.D., Leiberg S., Langner R., Kichic R., Hajcak G., Salkovskis P.M. (2002). The Obsessive-Compulsive Inventory: Development and validation of a short version. Psychol. Assess..

[39] Sica C., Ghisi M., Altoè G., Chiri L.R., Franceschini S., Coradeschi D., Melli G. (2009). The Italian version of the Obsessive Compulsive Inventory: Its psychometric properties on community and clinical samples. J. Anxiety Disord..

[40] Lovibond P.F., Lovibond S.H. (1995). The structure of negative emotional states: Comparison of the Depression Anxiety Stress Scales (DASS) with the Beck Depression and Anxiety Inventories. Behav. Res. Ther..

[41] Bottesi G., Ghisi M., Altoè G., Conforti E., Melli G., Sica C. (2015). The Italian version of the Depression Anxiety Stress Scales-21: Factor structure and psychometric properties on community and clinical samples. Compr. Psychiatry.

[42] Krueger R.F., Derringer J., Markon K.E., Watson D., Skodol A.E. (2012). Initial construction of a maladaptive personality trait model and inventory for DSM-5. Psychol. Med..

[43] Al-Dajani N., Gralnick T.M., Bagby R.M. (2016). A psychometric review of the Personality Inventory for DSM–5 (PID–5): Current status and future directions. J. Personal. Assess..

[44] Krueger R.F., Markon K.E. (2014). The Role of the DSM-5 Personality Trait Model in Moving Toward a Quantitative and Empirically Based Approach to Classifying Personality and Psychopathology. Annu. Rev. Clin. Psychol..

[45] Fossati A., Krueger R.F., Markon K.E., Borroni S., Maffei C. (2013). Reliability and validity of the Personality Inventory for DSM-5 (PID-5) predicting DSM-IV personality disorders and psychopathy in community-dwelling Italian adults. Assessment.

[46] Bottesi G., Ghisi M., Martignon A., Sica C. (2018). Self-other agreement in DSM-5 Section III Dimensional Personality Traits: A study on Italian community individuals. Personal. Individ. Differ..

[47] Kotov R., Krueger R.F., Watson D., Achenbach T.M., Althoff R.R., Bagby R.M., Brown T.A., Carpenter W.T., Caspi A., Clark L.A. (2017). The Hierarchical Taxonomy of Psychopathology (HiTOP): A dimensional alternative to traditional nosologies. J. Abnorm. Psychol..

[48] Cohen J. (1988). Statistical Power Analysis for the Behavioral Sciences.

[49] Hair J.F., Black W.C., Babin B.J., Anderson R.E., Tatham R.L. (2006). Multivariate Data Analysis.

[50] Brooks S.K., Webster R.K., Smith L.E., Woodland L., Wessely S., Greenberg N., Rubin G.J. (2020). The psychological impact of quarantine and how to reduce it: Rapid review of the evidence. Lancet.

[51] Luo M., Guo L., Yu M., Jiang W., Wang H. (2020). The Psychological and Mental Impact of Coronavirus Disease 2019 (COVID-19) on Medical Staff and General Public—A systematic Review and Meta-analysis. Psychiatry Res..

[52] Matsunaga H., Mukai K., Yamanishi K. (2020). Acute impact of COVID-19 pandemic on phenomenological features in fully or partially remitted patients with obsessive–compulsive disorder. Psychiatry Clin. Neurosci..

[53] Khosravani V., Asmundson G.J.G., Taylor S., Bastan F.S., Ardestani S.M.S. (2021). The Persian COVID stress scales (Persian-CSS) and COVID-19-related stress reactions in patients with obsessive-compulsive and anxiety disorders. J. Obsessive Compuls. Relat. Disord..

[54] Höfling V., Weck F. (2017). Hypochondriasis Differs from Panic Disorder and Social Phobia: Specific Processes Identified Within Patient Groups. J. Nerv. Ment. Dis..

[55] Noyes R., Stuart S., Watson D.B., Langbehn D.R. (2006). Distinguishing between hypochondriasis and somatization disorder: A review of the existing literature. Psychother. Psychosom..

[56] Rief W., Hessel A., Braehler E. (2001). Somatization symptoms and hypochondriacal features in the general population. Psychosom. Med..

[57] Norris L.A., Marcus D.K. (2014). Cognition in health anxiety and hypochondriasis: Recent advances. Curr. Psychiatry Rev..

[58] Tyrer P., Tyrer H. (2018). Health anxiety: Detection and treatment. BJPsych Adv..

[59] Lingiardi V., McWilliams N. (2017). Psychodynamic Diagnostic Manual: PDM-2.

[60] Kotov R., Ruggero C.J., Krueger R.F., Watson D., Yuan Q., Zimmerman M. (2011). New dimensions in the quantitative classification of mental illness. Arch. Gen. Psychiatry.

[61] Forbes M.K., Sunderland M., Rapee R.M., Batterham P.J., Calear A.L., Carragher N., Ruggero C., Zimmerman M., Baillie A.J., Lynch S.J. A Detailed Hierarchical Model of Psychopathology: From Individual Symptoms Up to the General Factor of Psychopathology. https://osf.io/3dp6f/.

[62] Hedman-Lagerlöf E. (2019). The Clinician’s Guide to Treating Health Anxiety: Diagnosis, Mechanisms, and Effective Treatment.

[63] Di Nardo M., Madonna M., Murino T., Castagna F. (2020). Modelling a Safety Management System Using System Dynamics at the Bhopal Incident. Appl. Sci..

[64] Golenia L., Schoemaker M.M., Otten E., Mouton L.J., Bongers R.M. (2017). What the Dynamic Systems Approach Can Offer for Understanding Development: An Example of Mid-childhood Reaching. Front. Psychol..

[65] Brown R.J., Skelly N., Chew-Graham C.A. (2020). Online health research and health anxiety: A systematic review and conceptual integration. Clin. Psychol..

[66] Hayes S.C., Strosahl K.D., Wilson K.G. (2011). Acceptance and Commitment Therapy: The Process and Practice of Mindful Change.

[67] Eilenberg T., Fink P., Jensen J.S., Rief W., Frostholm L. (2016). Acceptance and Commitment Group Therapy (ACT-G) for health anxiety: A randomized controlled trial. Psychol. Med..

